# Sub-cycle time resolution of multi-photon momentum transfer in strong-field ionization

**DOI:** 10.1038/s41467-019-13409-6

**Published:** 2019-12-05

**Authors:** Benjamin Willenberg, Jochen Maurer, Benedikt W. Mayer, Ursula Keller

**Affiliations:** Department of Physics, ETH Zurich, 8093 Zurich, Switzerland

**Keywords:** Atomic and molecular interactions with photons, Attosecond science

## Abstract

During multi-photon ionization of an atom it is well understood how the involved photons transfer their energy to the ion and the photoelectron. However, the transfer of the photon linear momentum is still not fully understood. Here, we present a time-resolved measurement of linear momentum transfer along the laser pulse propagation direction. We can show that the linear momentum transfer to the photoelectron depends on the ionization time within the laser cycle using the attoclock technique. We can mostly explain the measured linear momentum transfer within a classical model for a free electron in a laser field. However, corrections are required due to the parent-ion interaction and due to the initial momentum when the electron enters the continuum. The parent-ion interaction induces a negative attosecond time delay between the appearance in the continuum of the electron with minimal linear momentum transfer and the point in time with maximum ionization rate.

## Abstract (web summary)

Understanding momentum transfer in light–matter interaction is intriguing. Here the authors study momentum transfer from photons to electrons and observe a time delay in the linear momentum transfer during strong-field ionization of xenon atoms using time-resolved measurements with velocity map imaging.

## Introduction

Photon linear momentum transfer, that is momentum transfer along the laser beam propagation axis, upon the interaction of light with matter is one of the most fundamental processes in physics. It impacts a broad range of scientific fields, ranging from laboratory-scale photoionization experiments^1^ to plasma physics^2,3^, laser cooling of microscopic^4,5^ and macroscopic objects^6^ and it is the underlying mechanism for the occurrence of radiation pressure.

The simplest example for a process that involves transfer of linear momentum from a photon to an electron is Compton-scattering, where a photon scatters from a free electron. Whereas the fundamental concepts of energy and momentum conservation forbid the complete absorption of the photon by the free electron^7^, photons can be absorbed by a bound electron during photoionization.

In the case of single-photon ionization, the linear momentum of the photon ${E}_{{\rm{ph}}}/c$ with the photon energy ${E}_{{\rm{ph}}}$ ($c$ denotes the speed of light) is transferred to the electron–ion system along the laser propagation direction. For sufficiently high photon energies the complete linear momentum can be transferred to the outgoing electron^8,9^. And at low ionization potentials, the electron momentum can even exceed ${E}_{{\rm{ph}}}/c$^10–13^. In this case the ion receives a momentum in the opposite direction^8,10–13^.

The situation changes drastically if we consider photon energies well below the ionization potential of the target and high laser intensities. In this case, multiple photons are involved in the ionization process. So far, studies on linear momentum transfer from the laser field to either ions or photoelectrons investigated mainly the time-averaged final photoelectron momenta^13–18^ and the ionization-phase-dependent momentum transfers upon recollision^19,20^. However, to the best of our knowledge, there has been no experimental study on the time-dependent linear momentum transfer during photoionization – neither for single-photon nor for multi-photon ionization processes.

Here, we present a study on the time-resolved linear momentum transfer in strong-field ionization. Beyond the limit of the electric dipole approximation, we observe a time-dependent momentum transfer and we can show that the time-averaged photon radiation pressure picture is not generally applicable.

**Fig. 1:**
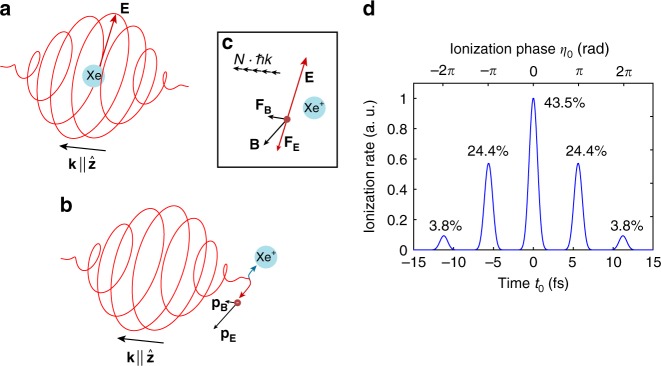
Timing of linear momentum transfer probed with the attoclock method. The rotating electric field vector serves as a time reference to clock the linear momentum transfer onto the electron. **a** The xenon atom at the time of ionization when the electric field is maximal. **b** The electron after the pulse has passed. The electron leaves the pulse at an angle of $\sim 9{0}^{\circ }\ (=\pi /2)$ with respect to the electric field vector at the time of ionization. Deviations from this angle are caused by the influence of the parent-ion interaction and ionization delay times. **c** Illustration of the forces acting on the outgoing electron after it was released from the ion. The magnetic field component of the laser field is responsible for a force in laser beam propagation direction. This force can be understood in terms of transfer of linear photon momentum onto the outgoing electron. **d** Ionization rate based on the ADK-theory^36^ during the pulse as function of the ionization phase ${\eta }_{0}$ as well as the time ${t}_{0}$.

We achieve sub-cycle time resolution on an attosecond scale by employing the attoclock method^21–24^. In this technique, the rotating electric field vector serves as reference for the timing of ionization processes. In our measurements we use the attoclock method to access the transferred linear momentum along the laser propagation direction as a function of the phase within a laser cycle at which the electron is released to the continuum. The measurement method is illustrated in Fig. 1. In a semi-classical picture the photoelectron is released to the continuum around the peak of the laser electric field (Fig. 1a). During the subsequent interaction with the electromagnetic pulse the electron with charge $-e$ is accelerated by the Lorentz force ${{\bf{F}}}_{{\bf{L}}}=-e\cdot ({\bf{E}}+{\bf{v}}\times {\bf{B}})$ (Fig. 1c) leading to a final momentum of the electron after the pulse with a component in the polarization plane ${{\bf{p}}}_{\perp }$ and in laser beam direction ${p}_{z}$ (Fig. 1b). In the multiphoton picture of the ionization process the ${p}_{z}$-drift of the electron corresponds to a partial transfer of photon linear momentum. Although strong-field ionization is in principle a multi-cycle process, the attoclock method allows us to access the dynamics within a cycle. In the polarization plane, the streaking angle reflects the ionization time, i.e. the point in time when the electron appears in the continuum, within a laser optical cycle (see Supplementary methods). The contribution from different cycles is shown for the case of zero carrier envelope phase (CEP) in Fig. 1d. For our experimental parameters the main contributions stem from the central cycle (43.5%) and the neighboring cycles (24.4% each).

We use the attoclock method to access the dependence of the total linear photon momentum that was transferred to the electron on the ionization time during the electromagnetic wave cycle with attosecond time resolution. In addition, we learn from our measurement that the parent–ion interaction induces a measurable negative attosecond time-delay between the photoelectron pathway with the highest probability and the one with the minimal linear photon momentum transfer. Furthermore, our study suggests that within the two-step model of strong-field ionization an additional initial momentum in laser beam propagation is required to explain our experimental results.

## Results and discussion

### Models of linear photon momentum transfer

To better understand the physics of linear momentum transfer in multi-photon strong-field ionization we first discuss a free electron that interacts with light. If the number of photons involved in the process is sufficiently high, the laser field can be described by a classical electromagnetic field. A widely used model for this situation is based on the classical theory of the high-intensity Thomson scattering, i.e. the low-energy limit of Compton scattering^25^: Governed by the laws of classical mechanics, the electron gets accelerated by the electric field of the light. If a free electron is passed by an intense classical light pulse, its final momentum is equal to its initial momentum once the laser pulse has completely vanished again. However, in the case when the free classical electron is born with an initial momentum ${{\bf{p}}}_{0}$ during the classical pulse, the laser field transfers kinetic energy to the electron. The dominant fraction of the momentum of the electron after the pulse is $\propto -{\bf{A}}({\eta }_{0})$ and directed in the polarization plane, i.e. the plane perpendicular to the propagation direction of the laser pulse. The transferred momentum that points along the beam propagation direction $z$ is a relatively small fraction of the overall momentum transfer and only non-zero beyond the electric dipole approximation.

The sudden appearance of a classical electron in a classical field of the laser pulse is one of the assumptions in the widely used semiclassical two-step models of strong-field ionization: The electron leaves the bound state with essentially zero momentum along the instantaneous negative electric field direction and is subsequently accelerated by the laser field^26–29^. The initial position and momentum of the electron are based on the laws of quantum mechanics. If the parent–ion interaction is neglected, the final momentum of the ionized photoelectron with the initial momentum ${{\bf{p}}}_{0}$ at a phase ${\eta }_{0}$ of the laser field can be calculated analytically: ${{\bf{p}}}_{{\rm{f}},\perp }={{\bf{p}}}_{0,\perp }-{\bf{A}}({\eta }_{0})$ is the final momentum component in the polarization plane, where ${{\bf{p}}}_{0,\perp }$ denotes the electron’s initial momentum in the polarization plane and ${\bf{A}}({\eta }_{0})$ the vector potential at the phase ${\eta }_{0}$ (atomic units are used throughout). In the propagation direction of the light, $z$, the final momentum is governed according to classical physics in the non-relativistic limit, however without the electric dipole approximation, by

1 $${p}_{{\rm{f}},z}={p}_{0,{\rm{z}}}+\frac{1}{2c}\ {\bf{A}}({\eta }_{0})\cdot ({\bf{A}}({\eta }_{0})-2\ {{\bf{p}}}_{0,\perp })$$

where ${p}_{0,{\rm{z}}}$ denotes the $z$ component of the electron’s initial momentum and $c$ the speed of light^30^ (for details see Supplementary Note 3). Since $| {\bf{A}}| \propto \lambda \cdot \sqrt{I}$ the non-dipole contributions can be observed either for high laser intensities $I$^31^ or long wavelengths $\lambda$^16,32^. In our experiment, the electron energies are in a regime, where the final momentum ${p}_{{\rm{f}},z}$ in beam direction is different from the initial ${p}_{0,{\rm{z}}}$.

Besides by the two-step model, strong-field ionization can in general be described in a photon picture as multi-photon above-threshold ionization^33^: A total number of ${N}_{{\rm{tot}}}={N}_{{\rm{sub}}}+{N}_{{\rm{above}}}$ photons is absorbed and the conserved photon linear momentum ${N}_{{\rm{tot}}}\cdot \hslash k$ is shared between the electron and ion^34^. Whereas the momentum ${I}_{{\rm{P}}}/c$ (${I}_{{\rm{P}}}$ the ionization potential of the target) of the ${N}_{{\rm{sub}}}$ photons needed to lift the electron into the continuum is rather transferred to the ion^15^, the quasi-free electron gets accelerated by the remaining ${N}_{{\rm{above}}}$ photons to its final kinetic energy ${E}_{{\rm{kin}}}$ with respect to the atomic core. Small offsets of the order of ${I}_{{\rm{P}}}/(3c)$ in the expectation value of the electron momentum in beam direction from the absorption of the ${N}_{{\rm{sub}}}$ photons have been theoretically predicted^13,35^. First experimental evidence for this ${I}_{{\rm{P}}}/(3c)$-shift was presented recently for the cycle-averaged case of circular polarization in the near-IR^18^. In contrast to all previous experimental studies on the topic of strong-field ionization beyond the dipole approximation that reported time-averaged expectation values of the final momentum distribution along ${p}_{z}$, we focus on the ${p}_{z}$ alteration of the PMD as a function of the ionization phase within the laser optical cycle.

### Subcycle-resolved ${p}_{z}$-shifts from 3D photoelectron momentum distributions (PMDs)

In our experiments, we use elliptically polarized mid-infrared (mid-IR) laser pulses centered around 3.4 μm to strong-field ionize the target xenon in a regime beyond the electric dipole approximation. The laser pulses were compressed based on the ionization signal to ~50 fs, corresponding at an optical cycle duration of 11.3 fs to a pulse duration of 4.4 cycles. The carrier envelope offset phase^37^ was not stabilized. At a repetition rate of 50 kHz we reach a peak intensity of $\sim 4\cdot 1{0}^{13}$ W cm^−2^. Accordingly, the Keldysh parameter is in the range $\gamma \approx 0.4 - 0.5$, an intermediate regime between pure multi-photon ionization ($\gamma \gg 1$) and pure tunnel ionization ($\gamma \ll 1$)^38^.

**Fig. 2:**
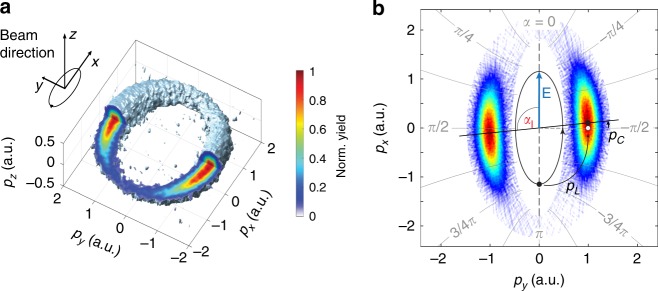
Momentum distributions from strong-field ionization with angular streaking. **a** Isosurface of a reconstructed 3D photoelectron momentum distribution (PMD) recorded at an ellipticity of $\epsilon =0.8$ together with a sketch of the polarization ellipse and the beam direction. **b** Polarization plane PMD for an ellipticity of $\epsilon$ = 0.5 visualizing the definition of elliptical coordinates and the streaking angle $\alpha$. Superimposed is the illustration of the effect of the parent–ion interaction onto an outgoing electron ionized at the peak of the electric field, as indicated by the polarization ellipse in black. The final momentum of the photoelectron gains in addition to the momentum ${\mathbf{p}}_{{\mathrm{{L}}}}$ acquired by the propagation in the laser field the momentum ${\mathbf{p}}_{{\mathrm{{C}}}}$ from the interaction with the parent ion mostly in the direction of the instantaneous electric field. The angle ${\alpha }_{{\mathrm{{I}}}}$ denotes the angle of maximal ionization yield.

Full 3D PMDs were recorded with a velocity map imaging (VMI) spectrometer in combination with a tomographic reconstruction algorithm^39,40^ (Fig. 2a). The studied ellipticities $\epsilon \ge 0.3$ do not allow for recollisions of the electron with the residual ion^20^.

From the 3D momentum distributions, we extracted the ionization yield as a function of an angle $\alpha$, defined in elliptical coordinates in the polarization plane (Fig. 2b). This ensures a linear mapping of time to angle also for comparably small ellipticites. From the resulting photoelectron angular distribution, we identified an angle ${\alpha }_{{\rm{I}}}$, where the ionization yield maximizes. Furthermore, from radially integrated 3D momentum distributions we extracted the shift in ${p}_{z}$-direction as a function of $\alpha$ and identified the angle ${\alpha }_{{\rm{M}}}$, where the momentum transfer from the laser field onto the photoelectrons minimizes. The details for the data analysis are described in the Supplementary methods and Supplementary Fig. 2. We observe that the electrons ionized around the maximal electric field experience the smallest shift in ${p}_{z}$.

In the multi-photon picture of the strong-field ionization process the ${p}_{z}$ shift is a direct measure for the number of photons that transferred their linear momentum to the electron. Our measurement for elliptical polarization shows that the number of involved photons varies within an optical cycle. Specifically, for the electrons born around the electric field maximum the number of absorbed photon momenta is smaller than the prediction from the radiation pressure model: Our experiment with ellipticity $\epsilon =0.5$ shows a minimal ${p}_{z}$ shift of $\approx 5\cdot 1{0}^{-3}$ a.u. corresponding to $\sim$50 photon momenta. The radiation pressure model would predict an average shift of ${U}_{{\rm{P}}}/c\approx 1\cdot 1{0}^{-2}$ a.u. corresponding to $\sim$100 photon momenta. ${U}_{{\rm{P}}}$ is the ponderomotive energy of the photoelectron in the laser field.

This shows that the radiation pressure picture, used to explain the linear momentum transfer in the case of circular polarization^15^, is not generally applicable for arbitrary polarization states. For elliptical and linear polarization, it is not even generally applicable for the cycle-averaged photelectron momentum $\left\langle {p}_{z}\right\rangle$ due to weighting of ${p}_{z}$ with the nonlinear ionization rate during the laser cycle. The radiation pressure picture applies only if the magnetic force onto the electron is constant over one full laser cycle, i.e. for purely circular polarization.

### Attosecond time delays between the most probable electron pathway and the one with minimal ${p}_{z}$-shift

**Fig. 3:**
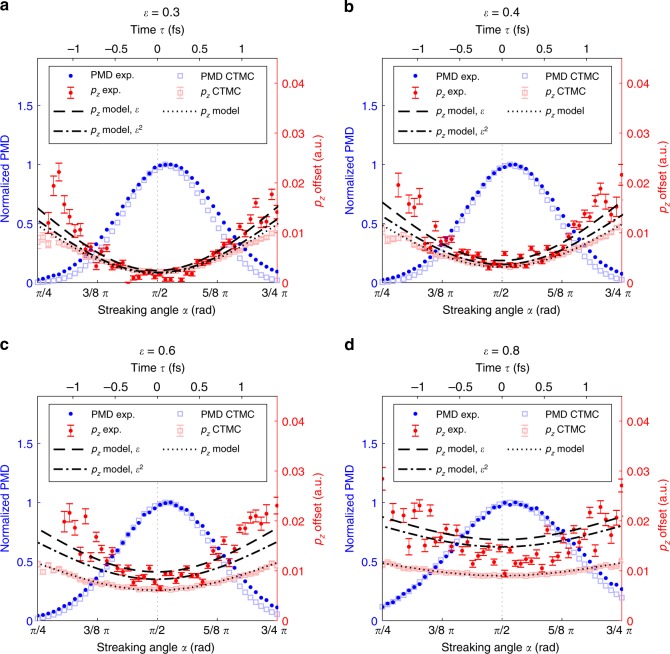
Sub-cycle dependence of linear momentum transfer. Angular distributions for various ellipticities of the ${p}_{z}$-shift (red) together with the normalized electron yield (blue) from the experimental data (PMD exp., ${p}_{z}$ exp.). In addition, we show the corresponding results from our classical trajectory Monte-Carlo (CTMC) simulations (PMD CTMC, ${p}_{z}$ CTMC) and the theoretical prediction for a peak intensity of ${I}_{0}=4\cdot 1{0}^{13}\ {\rm{W}}\ {{\rm{cm}}}^{-2}$ based on a classical model (${p}_{z}$ model) and an extended version with an additional initial momentum component along ${p}_{z}$ (${p}_{z}$ model, $\epsilon$ and ${p}_{z}$ model, ${\epsilon }^{2}$) (for details see main text, Supplementary Notes 2–5 and Supplementary discussions). The error bars are of purely statistical nature and are based on the $1\sigma$-uncertainty from the gaussian fit used to extract the ${p}_{z}$-shift for each angular step.

The variation of the ${p}_{z}$-shift as a function of the streaking angle $\alpha$, i.e. the ionization phase ${\eta }_{0}$, can be largely explained by the previously introduced model for a free electron (Eq. (1)). The model predicts a minimum of the ${p}_{z}$-shift around ionization phase ${\eta }_{0}=0$ (see Supplementary Notes 2–4, Supplementary Figs. 4–6) and the correct order of magnitude for the modulation of $\left\langle {p}_{z}\right\rangle$ as a function of $\alpha$, including a decrease of the $\left\langle {p}_{z}\right\rangle (\alpha )$ variation amplitude with increasing ellipticity (Fig. 3).

**Fig. 4:**
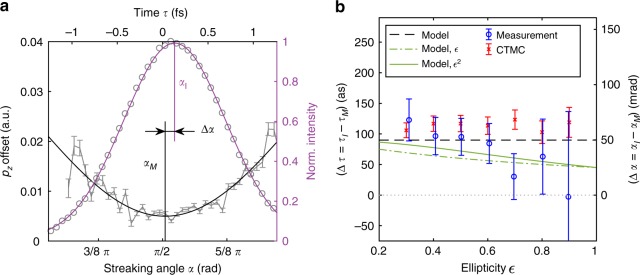
Attosecond timing of linear momentum transfer. **a** Visualization of the angle difference between the angles where ${p}_{z}$ minimizes (${\alpha }_{{\mathrm{{M}}}}$) and the ionization yield maximizes (${\alpha }_{{\mathrm{{I}}}}$) for an ellipticity of $\epsilon =0.5$. The corresponding time axis up to a constant shift is shown on top. **b** Angular difference $\Delta \alpha$ as a function of the ellipticity, together with the corresponding time difference axis. The error bars are based on the 1$\sigma$ uncertainty of the fit. The measurement is compared to the CTMC calculations, and three analytic models for a free electron corrected by the electron–ion interaction (Model) and extended additionally by an initial momentum along ${p}_{z}$ (Model $\epsilon$ and Model ${\epsilon }^{2}$) (for details see main text and supplementary methods).

In the experiment the extracted angles ${\alpha }_{{\rm{M}}}$ and ${\alpha }_{{\rm{I}}}$ for minimal ${p}_{z}$-shift and maximal PMD signal, respectively, show a positive offset $\Delta \alpha ={\alpha }_{{\rm{I}}}-{\alpha }_{{\rm{M}}}$ for all ellipticities (Fig. 4, details see Supplementary Methods and Supplementary Fig. 2). The measured angle differences are small but self-referenced to the peak of the ionization yield, which cancels systematic error sources. We apply the attoclock principle to translate the angular offset into a time offset. The usage of an elliptical coordinate system in the polarization plane results in a linear mapping between the streaking angle $\alpha$ and the time $\tau$ or ionization phase ${\eta }_{0}$ (see Supplementary methods). When we translate $\Delta \alpha$ to a time offset $\Delta \tau$, we find that the electrons with a minimal ${p}_{z}$-shift are released into the continuum before the maximum of the electric field on the order of 100 as for small ellipticities assuming that the maximal ionization yield corresponds to the maximum of the electric field.

Under the assumption of zero initial momentum, the simple model for a free electron predicts a vanishing offset $\Delta \alpha$. Both electron trajectories, the most likely one and the one experiencing the smallest linear momentum transfer would be ionized at identical phase ${\eta }_{0}=0$.

The more complete description of the continuum propagation in our classical trajectory Monte-Carlo (CTMC) simulations covers the interaction of the photoelectron with the residual ion. As a result the CTMC simulations predict in addition to the ionization phase-dependent $\left\langle {p}_{z}\right\rangle$ (Fig. 3) a negative ionization phase for the minimal ${p}_{z}$-shift. This results in a positive $\Delta \alpha$ of the same order of magnitude as found in the experiment (Fig. 4b).

We include the interaction with the residual ion in the analytic model perturbatively by setting the initial photoelectron momentum to ${{\bf{p}}}_{0}={{\bf{p}}}_{{\rm{C}}}$. This is based on the fact that the final momentum can be decomposed in the polarization plane into ${{\bf{p}}}_{{\rm{f}}}={{\bf{p}}}_{{\rm{L}}}+{{\bf{p}}}_{{\rm{C}}}$, where ${{\bf{p}}}_{{\rm{L}}}$ is the momentum transferred onto the electron by the laser field and ${{\bf{p}}}_{{\rm{C}}}$ is the momentum acquired by the Coulomb interaction with the residual ion^41^ (see Supplementary Note 5). The analytic solution for the ionization phase ${\eta }_{0}$ with minimal ${p}_{z}$,

2 $${\eta }_{0}=-0.5\ \arctan \left(\frac{\pi }{\sqrt{2}}\ \omega \ {I}_{{\rm{P}}}^{-3/2}\right)\ ,$$

is independent of the laser field intensity and ellipticity $\epsilon$. The Coulomb-corrected analytic predictions are in perfect agreement with the numerical predictions from the CTMC calculations (compare Figs. 3 and 4). This shows that the Coulomb interaction is mostly responsible for the angle offset $\Delta \alpha$ and the corresponding time offset.

However, the measured absolute values for $\left\langle {p}_{z}\right\rangle$ lie slightly above the values expected from both, our CTMC calculations and the analytical model. The deviation can be partly caused by the combined influence of the focal volume averaging and an uncertainty in the intensity calibration and the determination of the absolute zero momentum of the detector. The error bars shown in Fig. 3 are of statistical nature based on the fit and do not include any of the possible systematic errors. Neither of the above can fully explain the discrepancy in the curvature of the ${p}_{z}$-shift as a function of the streaking angle. Moreover, the deviation in the linear momentum transfer (compare Supplementary Fig. 3 and Supplementary discussions) and the experimentally measured delay times $\Delta \tau$ appear to be ellipticity dependent, whereas the CTMC-simulations and the analytic model do not predict a ellipticity dependence of the time offset (Fig. 4b).

We suggest to introduce an additional initial momentum along the beam propagation direction that varies with the ionization phase $\eta$ as $\frac{1}{2c}| {\bf{A}}(\eta ){| }^{2}$. This correction is based on the photon momentum corresponding to the additional energy of the electron in the laser field described by the vector potential $A(\eta )$ when it appears in the continuum (see Supplementary discussion). In addition, our data suggests an ellipticity-dependent initial momentum of $\frac{1}{2c}f(\epsilon )| {\bf{A}}(\eta ){| }^{2}$, where $f(\epsilon )$ is a non-constant function of the ellipticity $\epsilon$. The additional linear momentum at the tunnel exit improves the agreement between the semiclassical model and the experimental observations in two aspects for the exemplary functions $f(\epsilon )=\epsilon$ and $f(\epsilon )={\epsilon }^{2}$: The absolute value of the linear momentum transfer in beam direction ${p}_{z}$ as a function of the streaking angle becomes less underestimated (Fig. 3). For the ellipticity-dependent prefactor $f(\epsilon )$ the ionization phase difference between the most likely electron and the one corresponding to the minimal linear momentum transfer decreases with increasing ellipticity (Fig. 4b).

In conclusion, we experimentally demonstrated that the strong-field momentum transfer in laser propagation direction from the field onto the photoelectrons beyond the limit of the dipole approximation is a time-dependent process within an optical cycle. Thus, a time-averaged radiation pressure picture is not applicable in the general case of elliptical polarization. The time-dependent momentum transfer in strong-field ionization can mostly be explained with a classical model of a free electron, extended by the parent–ion interaction of the escaping photoelectron and an additional time-dependent initial momentum shift related to the energy of an electron in an electromagnetic field at the phase of ionization ${\eta }_{0}$. The shift of the PMD along the laser propagation direction provides direct access to the number of photons that were absorbed by the electron during the ionization process. Hereby, our results open-up possibilities for measurements on the timing of photon absorption, as well as fluctuations of the laser field^42^.

More specifically, we observed a time delay between the photoelectron emission times of maximal ionization yield and minimal linear momentum transfer. As the ionization rate is connected to the cycles of the laser field, our observations imply a time delay between the cycles of the field and the linear momentum transfer. We showed that the electrons with the smallest momentum transfer are ionized shortly before the peak of the electric field with a time delay on the order of several tens of attoseconds (Fig. 4). This time delay might contain further information about the electron dynamics in the classically forbidden region during the ionization process.

Our findings have important consequences for all areas of physics that are influenced by the field-momentum transfer. They also trigger the question about time delays in the field-momentum transfer in the case of single photon ionization, i.e. if the parent–ion potential induced time delays are a general property of photoionization or apply only for the case of strong-field ionization. Thus, our results can motivate further studies with single photon ionization via time-dependent ${p}_{z}$-transfer in streaking and RABBITT experiments.

## Methods

### Laser system

We use an optical parametric chirped pulse amplifier system that delivers ultrashort laser pulses at a center wavelength of 3.4 μm with a pulse duration of 50 fs, a pulse energy of up to 20 μJ at a repetition rate of 50 kHz^43,44^ and with random CEP^37^.

The polarization state of the laser beam is fully controlled with a broadband custom-made quarter-wave plate and a subsequent half-wave plate (B. Halle). The first waveplate controls the ellipticity and the second one the orientation of the polarization ellipse. Both waveplates were fully characterized by optical polarimetry measurements (see Supplementary methods).

### VMI spectrometer

We use a VMI setup in the classical configuration, that consists of three electrodes^45,46^ to detect the strong-field-ionized photoelectrons. The electrostatic lens system projects the generated photoelectrons in momentum space onto the detector plane. The photoelectron signal is detected by a microchannel plate (MCP) backed by a phosphor screen that is read out by a low noise CCD camera (PIXIS Princeton Instruments). We tightly focus the laser beam in a back-focusing mirror geometry ($f=1.5$ cm) into our high-density gas target. Due to the short focal length of the mirror it is placed in between the repeller and extractor plate of the spectrometer. The diameter (3 cm) of the hole in the extractor plate is such that the mirror does not enter the free-imaging volume of the spectrometer. Reference measurements for strong-field ionization at 800 nm have shown no significant asymmetry of the PMD in laser beam propagation direction. This shows that the spectrometer field does not get distorted by the dielectric mirror.

### Three-dimensional PMDs

With the elliptically polarized 50 fs, 3.4 μm mid-IR laser pulses from our OPCPA system we ionize xenon at peak intensities of $4\cdot 1{0}^{13}$ W cm^−2^. We recorded two-dimensional (2D) projections of the three-dimensional (3D) PMD perpendicular to the polarization plane with a VMI spectrometer in steps of 1° covering a range of 180° by the rotation of the polarization ellipse. The rotation axis coincides with the laser beam propagation direction. The orientation of the PMD in the VMIS is rotated due to the rotation of the polarization since both are linked one to one. From the set of projections we recover the full 3D momentum distribution with a tomographic reconstruction algorithm (filtered back projection)^39,40,47^. We apply a Hamming filter in the Fourier domain to suppress high-frequency noise that might lead to instabilities in the reconstruction. The cutoff of the filter is set to 0.4 times the Nyquist frequency for binning of the momentum space with a bin width of $5\cdot 1{0}^{-3}$ a.u.

### Intensity calibration

For the intensity calibration, we compared the radial distribution of reference measurements recorded with circular polarization with our CTMC calculations, a common method to determine the peak intensity in strong-field experiments^23,48^.

We also compared the short axis of the polarization ellipse in the polarization plane of the PMD from the full tomographic measurements with our CTMC calibration data and find the same peak intensities in agreement with the circular reference images.

A well-known inaccuracy of this method is caused by non-zero initial longitudinal momentum of the electron as the adiabatic approximation is not fully valid at near-IR wavelengths and low intensities^24,38,49^. However, under the presented experimental conditions, the adiabatic approximation is sufficiently valid. We estimate the deviation due to the adiabatic approximation to be 2–3% in the field and accordingly <0.1% in intensity^49^.

### CTMC simulations

We compare our experimental results with CTMC simulations. The starting conditions (ionization time, tunnel exit and momenta) for the classical trajectories are calculated from tunnel ionization theory in parabolic coordinates. The photoelectrons are liberated into the continuum according to the ADK ionization rate depending on the instantaneous magnitude of the electric field vector ${\bf{E}}$^36^.

The initial position of the electron trajectory at the tunnel exit is calculated based on the TIPIS model^48^. The tunnel exit is located at a distance

$${r}_{0}=\frac{1}{2}\left[{I}_{{\rm{P}}}+\sqrt{{I}_{{\rm{p}}}^{2}-4| {\bf{E}}| (1-\sqrt{2{I}_{{\rm{P}}}}/2)}\right]/| {\bf{E}}|$$

from the residual ion core in the negative direction of the instantaneous laser electric field. ${I}_{{\rm{P}}}={I}_{{\rm{P,0}}}+0.5({\eta }_{{\rm{A}}}-{\eta }_{{\rm{I}}})| {\bf{E}}{| }^{2}$ is the stark-shifted ionization potential of the target, in our case xenon with ${I}_{{\rm{P,0}}}=12.13\,{\rm{eV}}$, ${\eta }_{{\rm{A}}}=27.66\,\rm{a{.}u.}$ and ${\eta }_{{\mathrm{{I}}}}=20.0\,\mathrm{a{.}u.}$ are the static polarizability of the atom (Xe) and ion (Xe^+^), respectively. The initial longitudinal momentum at the tunnel exit in the direction of the instantaneous electric field vanishes. The initial momentum transverse to the instantaneous electric field is symmetrically Gaussian distributed according to the TIPIS model.

In the simulations we use a Gaussian pulse with a duration $\tau =50$ fs (FWHM in intensity), a center wavelength of 3.4 μm and a freely running CEP in the interval $[0,2\pi ]$.

After the ionization step the dynamics of the photoelectron is described in the classical continuum by solving Newton’s equations of motion including the Coulomb potential of the residual ion and the laser magnetic and electric fields. In the calculation we include the static polarizability of the residual ion caused by the instantaneous electric field of the driving laser pulse. The position of the ion (that is heavy compared to the mass of the non-relativistic photoelectron) is fixed at the origin of the coordinate system (lab frame). Any electron–electron correlation effects are fully neglected.

To sample the starting conditions (ionization time and CEP) we use importance sampling with flat prior in the CEP variable and gaussian in the ionization time variable. Weights are calculated according to the ionization rate.

We use the adaptive ODE solver implementation of 7/8 order based on the Runge–Kutta–Fehlberg method with relative and absolute error limit set to $1{0}^{-12}$. After propagation to the end of the laser pulse (${t}_{\max }=5\tau$) we apply Kepler’s formula for the mapping to asymptotic momenta^50,51^.

For sufficient statistics we simulate $1\cdot 1{0}^{8}$ trajectories for each fixed parameter set (peak intensity, ellipticity).

Trajectories ending with a negative total energy at the end of the pulse, i.e. bound Rydberg state-like trajectories, are discarded. The fraction of trajectories captured in a Rydberg state is basically zero ($<1{0}^{-5}$ for $\epsilon =0.3$) and decreases with increasing ellipticity.

## Supplementary information

Supplementary information

## Data Availability

The data that support the plots within this paper and other findings of this study are available from the corresponding author upon reasonable request.
