# Supplementary material for: Sub-cycle time resolution of multi-photon momentum transfer in strong-field ionization
Source: Nat Commun. 2019 Dec 5;10:5548. doi: 10.1038/s41467-019-13409-6 (PMC6895185; doi:10.1038/s41467-019-13409-6)
Supplement: Supplementary file 1 — Supplementary information [file 41467_2019_13409_MOESM1_ESM.pdf]

Supplementary information

Sub-cycle time resolution of multi-photon momentum transfer in strong-field ionization

Willenberg *et al.*

Contents

Supplementary Figures **3**

Supplementary Notes **9**

1 General remarks about strong-field ionization 9

2 Single trajectory calculations 10

3 Analytic model of a free electron in a laser field 10

4 Comparison to free electron model within the electric dipole approximation 12

5 Perturbative treatment of the electron-ion Coulomb interaction 12

Supplementary Discussions **15**

Supplementary Methods **17**

Nomenclature

(p_α, p_ρ, p_z) linear momentum of the photoelectron in elliptical coordinate representation

(p_x, p_y, p_z) linear momentum of the photoelectron in Cartesian coordinate representation

α streaking angle in the polarization plane in elliptical coordiantes

ϵ ellipticity of the laser field

η phase of the laser field

γ Keldysh parameter

$\mathbf{A}(\eta)$ vector potential of the laser field

I_{P} ionization potential

p_z linear momentum of the photoelectron in laser beam direction

U_{p} ponderomotive energy

ADK Ammosov Delone Krainov model for strong-field ionization

CEP carrier envelope phase

CTMC classical trajectory Monte-Carlo (simulations)

MCP microchannel plate

mid-IR mid-infrared

ODE ordinary differential equation

PMD photoelectron momentum distribution

RABBITT reconstruction of attosecond beating by interference of two-photon transitions

VMIS velocity map imaging spectrometer

Supplementary Figures

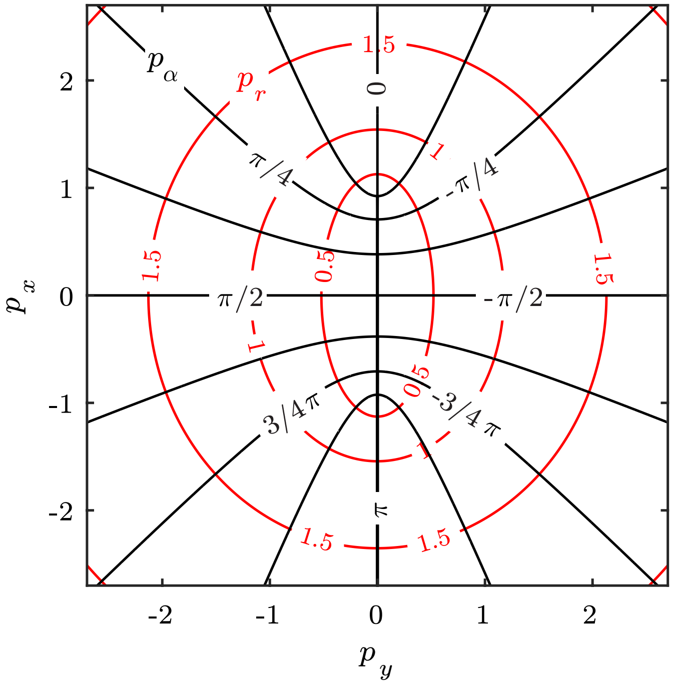

Supplementary Figure 1: Elliptical coordinate system. Illustration of the isolines for the two coordinates of the elliptical coordinate system. The isolines for the angle coordinate α are shown as black lines and the isolines for the radial coordinate ρ are shown as black lines.

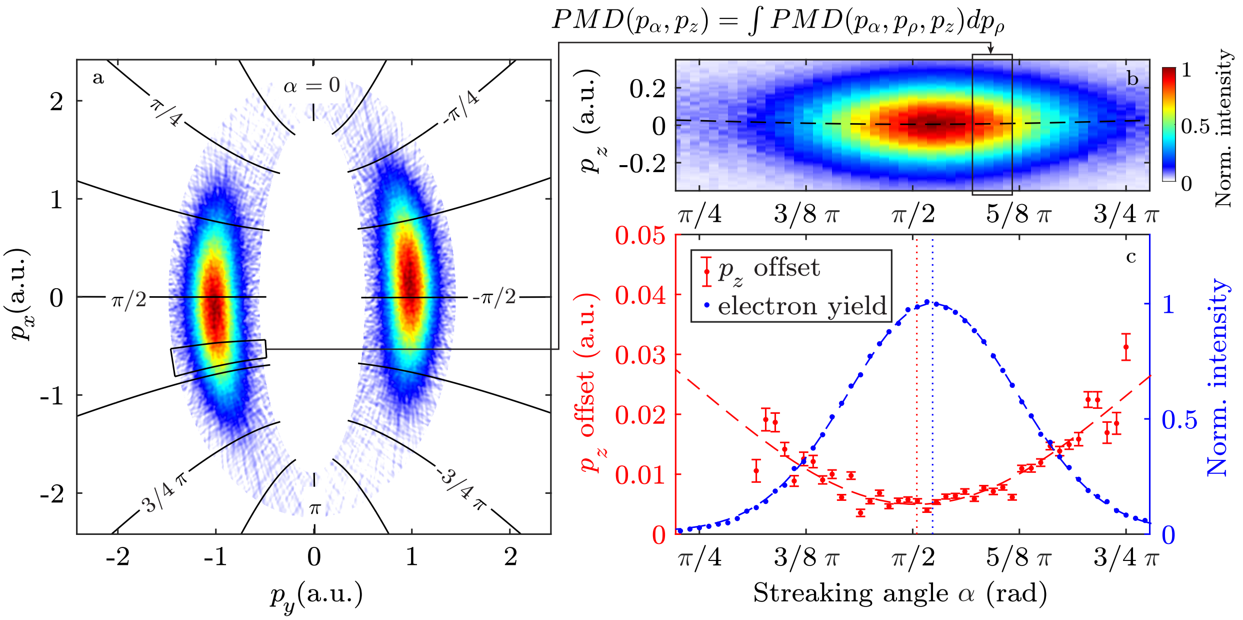

Supplementary Figure 2: Illustration of the data analysis flow. (a) Polarization plane cut of a PMD with the isolines of the α-coordinate laid over. (b) A plot of the α-z distribution after 'radial' integration over ρ. A section in the polarization plane and the corresponding section in the p_α-p_z momentum distribution are highlighted by black boxes. (c) Plot of the extracted p_z-offset (red) and the p_z-offset vs the angular streaking coordinate α. The error bars are based on the 1σ-deviation from the gaussian fit for each bin in α. For details see text.

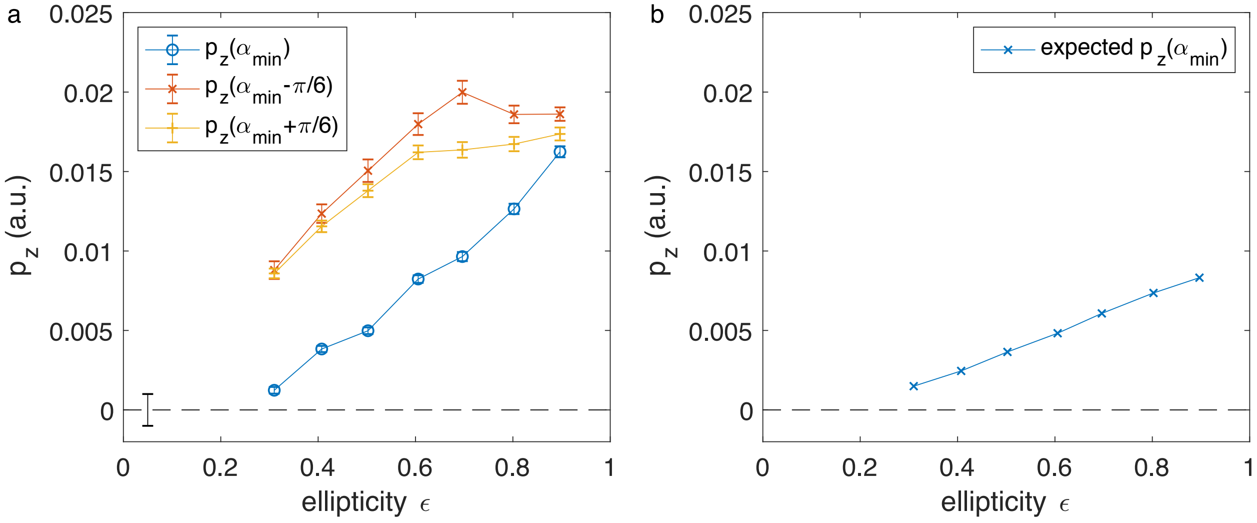

Supplementary Figure 3: Ellipticity-dependence of the linear photon momentum. (a) Measured linear momentum transfer in laser beam direction p_z for streaking angle α_{min} with minimal value p_z^{min} and at streaking angle $\alpha_{\mathrm{min}} \pm \pi/6$ as a measure for the ϵ-dependence of the p_z-variation, $p_z(\alpha_{\mathrm{min}} - \pi/6)$ and $p_z(\alpha_{\mathrm{min}} + \pi/6)$, respectively. The error bars correspond to the 1σ confidence interval of the fit to the p_z versus α curves. On the zero line we show the uncertainty due to systematic errors in the zero momentum calibration. (b) Expected linear momentum transfer calculated according to supplementary equation (7) from the short axis of the PMD in the polarization plane.

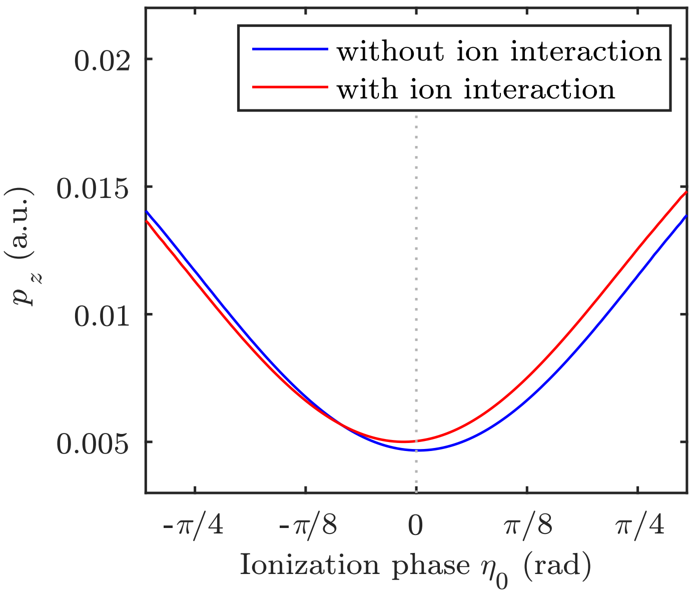

Supplementary Figure 4: Single trajectory calculations for xenon. We show the transferred linear momentum p_z vs the phase η_0 when it was released into the continuum for an ellipticity of $\epsilon = 0.5$ and a peak intensity of $4 \times 10^{13}\,\mathrm{W\,cm^{-2}}$. The electron is released with an initial momentum of $\mathbf{p_0} = 0$ at a position that reflects the tunnel exit in parabolic coordinates. The plot shows that the influence of the parent-ion Coulomb interaction shifts the release phase for the electron trajectory with the minimum p_z towards lower values.

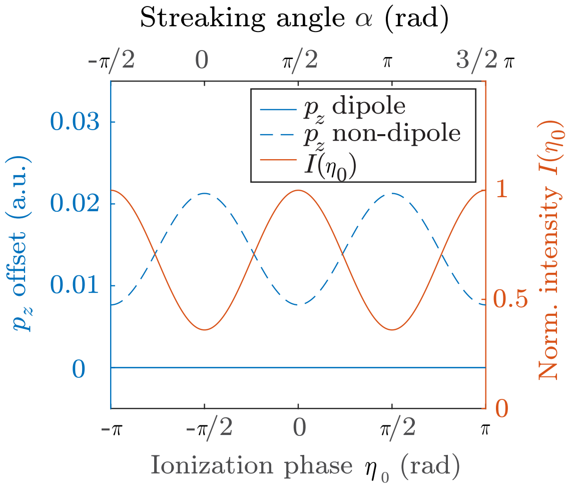

Supplementary Figure 5: Simple model for sub-cycle resolved linear momentum transfer. We show the classical expectation (dashed line, eq. 3) of the angle-resolved shift in p_z-direction for a single free electron born during an elliptical polarized laser pulse ($\epsilon = 0.5$) with zero initial momentum and neglecting the interaction with the residual ion. The flat solid line shows the expected zero p_z-offset within the dipole approximation. In addition, we show the dependence of the laser intensity on the phase of the laser field.

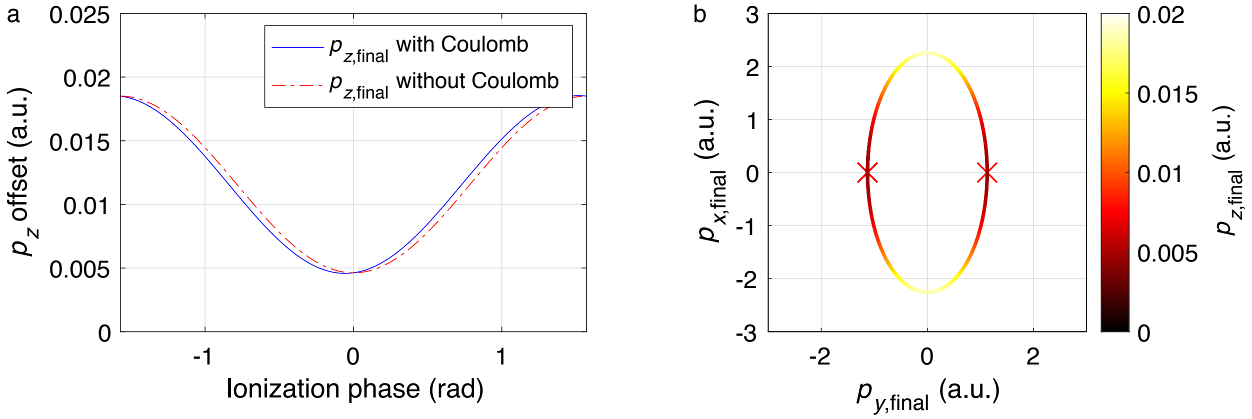

Supplementary Figure 6: Final photoelectron momenta from simple classical model. We show the final momenta from simple single-trajectory classical model of strong-field ionization of xenon at an intensity of $I_0 = 4 \times 10^{13}\,\mathrm{W\,cm^{-2}}$ and an ellipticity $\epsilon = 0.5$. (a) Final momentum of the photoelectron p_z as a function of the ionization phase neglecting the effect of the Coulomb potential of the residual ion and including the effect of the Coulomb potentenial perturbatively (detail see text). (b) Polarization plane representation of the final photoelectron momentum including the effect of the Coulomb potential. The p_z momentum in laser beam propagation direction is color-coded. The two red crosses mark the position of minimal linear momentum transfer in beam propagation direction.

Supplementary Notes

1 General remarks about strong-field ionization

Strong-field ionization is typically described in two different pictures: multiphoton ionization and tunnel ionization. The parameter space where these two pictures are applicable are typically characterized by the Keldysh parameter $\gamma = \sqrt{I_\mathrm{P}}/\sqrt{2U_\mathrm{p}}$ where I_P denotes the ionization potential of the target and U_p the ponderomotive potential in atomic units (used throughout if not stated otherwise). Traditionally, for $\gamma \ll 1$, the tunnel ionization model is applied and for $\gamma \gg 1$ a multiphoton picture is applied.

Multiphoton picture. In the multiphoton picture, the electron is lifted off the ionization potential by several photons and is accelerated by the absorption of additional photons. The absorbed photons have to provide the energy to overcome the effective ionization potential I_p and, in addition, the ponderomotive potential U_p, since the electron is initially released into the laser field. However, the additional energy that is necessary to overcome U_p is for short (i.e. sub-picosecond) laser pulses transferred back to the laser field instead of being transferred to kinetic energy of the photoelectron after the pulse. Thus, for sufficiently short pulses the electron has the final energy:

$$E_{kin}^{e^-} = N_\mathrm{tot} \cdot \hbar\omega - I_\mathrm{p} - U_\mathrm{p}$$

The transferred momentum onto the electron-ion system after the pulse has passed is shared between the ion and the electron. To date studies have suggested that the linear momentum of $E_\mathrm{kin}^{e^-}/c$ is transferred to the electron whereas the momentum of I_P/c is transferred to the ion [1]. The momentum of I_P/c corresponds to the number of photons that are necessary to lift the electron above the effective ionization potential. Deviations on the order of $\frac{I_\mathrm{P}}{3c}$ have been reported in theoretical studies [2,3].

Semiclassical two-step model. A complementary approach to describe a strong-field ionization process is the semiclassical two step model of strong-field ionization [4–7]. In this model, the electron is released into the continuum via the quantum mechanical tunnel ionization process and afterwards

streaked classically in the laser field, or, depending on the level of sophistication of the model, in the combined laser field and parent-ion potential. Before the ionization process, when a hydrogen-like atom is field-free, the electron has the total energy $E_{\mathrm{tot}} = -I_{\mathrm{P}}$.

Validity of the adiabatic approximation. The validity of the adiabatic tunnel ionization that serves as basis for the two-step model of strong-field ionization has been questioned [8,9]. However, at a mid-IR wavelength of 3.4 μm the non-adiabatic deviations from the adiabatic model are sufficiently small [10] to be neglected. One of the issues typically discussed is that the radial momentum distribution is affected by the influence of non-adiabaticity. In our case, we estimate the influence onto the relative final momentum of the electron to be smaller than 2 % [11].

2 Single trajectory calculations

We can illustrate the influence of the parent-ion interaction with the outgoing electron onto the phase η_0 when the electron is released into the continuum with single trajectory calculations. This has the advantage that one final p_z-component can be directly related to one initial phase η_0 without any fitting procedure. For the single trajectory calculations the electron starts at a phase η_0 with the initial momentum $\mathbf{p}_0 = 0$. The final momentum p_z versus emission phase η_0 curves with and without parent-ion interaction included (Fig. 4) clearly illustrate that the parent ion interaction leads to a reduced ionization phase η_0 for the electron trajectory with minimal final p_z. The smaller value of the phase, translated to time, means that the electron trajectory with the lowest linear momentum transfer is released prior to the peak of the electric field within the optical cycle, where the ionization probability is the highest.

3 Analytic model of a free electron in a laser field

In this section we derive the final momentum of a free electron that appears in the continuum during a laser pulse at the phase η_0. Let $\mathbf{A} = f(t) \cdot \mathbf{A}(\eta)$ with $\eta = kz - \omega t$ be the vector potential describing the laser pulse propagating in positive z direction with center frequency ω, i.e. wavelength $\lambda = 2\pi c/\omega$. In the notation for the following derivation we will drop the pulse

envelope $f(t)$ and simply assume, that $\lim_{\eta\to\infty} \mathbf{A}(\eta) = 0$.

We use the Hamilton formalism to address the problem. The non-relativistic Hamiltonian for a free particle with charge e (not necessarily the elementary charge) and mass m in an electromagnetic field described by the vector potential $\mathbf{A}$ and the scalar potential ϕ is given by

$$H = \sum_i \frac{(P_i - eA_i)^2}{2m} + e\phi\,. \tag{1}$$

$P_i = m\dot{x}_i + eA_i$ are the canonical momenta of the positions x_i. For the following derivation of the final momentum, we consider only a pure vector field ($\phi = 0$) without any scalar potential (like the Coulomb-potential). We obtain from the two Hamilton equations

$$\frac{dP_i}{dt} = -\frac{\partial H}{\partial x_i} \quad \text{and} \quad \frac{dx_i}{dt} = \frac{\partial H}{\partial P_i}$$

that the canonical momenta in the polarization plane (x-y-plane) are conserved:

$$\begin{aligned} \frac{dP_x}{dt} &= -\frac{\partial H}{\partial x} = -\frac{\partial}{\partial x}\left(\sum_i \frac{(P_i - eA_i(\eta(z,t)))^2}{2m}\right) = 0 \\ \frac{dP_y}{dt} &= -\frac{\partial H}{\partial y} = -\frac{\partial}{\partial y}\left(\sum_i \frac{(P_i - eA_i(\eta(z,t)))^2}{2m}\right) = 0\,. \end{aligned}$$

Further, we calculate the action of the electromagnetic field on the z component of the canonical momentum P_z. From the first Hamilton equation we obtain:

$$\begin{aligned} \frac{dP_z}{dt} &= -\frac{\partial}{\partial z}\left(\sum_i \frac{(P_i - eA_i)^2}{2m}\right) = -\frac{1}{2m}\sum_i 2(P_i - eA_i)(-e)\frac{\partial A_i}{\partial z} \\ &= \frac{e}{m}(\mathbf{P} - e\mathbf{A})\cdot\frac{\partial \mathbf{A}}{\partial z} = -\frac{e}{mc}(\mathbf{P} - e\mathbf{A})\cdot\frac{\partial \mathbf{A}}{\partial t}\,. \end{aligned}$$

For the last step we use, that $\frac{\partial \mathbf{A}}{\partial t} = \frac{\partial \mathbf{A}}{\partial \eta}\frac{\partial \eta}{\partial t} = -\omega\frac{\partial \mathbf{A}}{\partial \eta}$, $\frac{\partial \mathbf{A}}{\partial z} = \frac{\partial \mathbf{A}}{\partial \eta}\frac{\partial \eta}{\partial z} = k\frac{\partial \mathbf{A}}{\partial \eta}$ and $\omega/k = c$. Integrating the equation from the release time t_0 of the photoelectron until the end of the pulse, i.e. to $+\infty$, we obtain the final canonical momentum $P_{z,f}$ of the photoelectron:

$$\begin{aligned} P_{z,\mathrm{f}} - P_{z,0} &= \int_{t_0}^{\infty} \frac{dP_z}{dt}dt = -\frac{e}{mc}\left\{\int_{t_0}^{\infty} \mathbf{P}\cdot\frac{\partial \mathbf{A}}{\partial t}dt - e\int_{t_0}^{\infty} \mathbf{A}\cdot\frac{\partial \mathbf{A}}{\partial t}dt\right\} \\ &= -\frac{e}{mc}\left\{[\mathbf{P}\cdot\mathbf{A}]_{t_0}^{\infty} - \int_{t_0}^{\infty}\frac{\partial \mathbf{P}}{\partial t}\cdot\mathbf{A}\,dt\right\} + \frac{e^2}{2mc}\left[\mathbf{A}^2\right]_{t_0}^{\infty} \\ &= \frac{e}{mc}\mathbf{P}_0\cdot\mathbf{A}(\eta_0) - \frac{e^2}{2mc}\mathbf{A}(\eta_0)\cdot\mathbf{A}(\eta_0)\,. \end{aligned}$$

The integral with integrand $\frac{\partial \mathbf{P}}{\partial t} \cdot \mathbf{A}$ vanishes because the canonical momentum is conserved in the polarization plane and the vector potential is identical to zero in beam propagation direction z (plane wave), i.e. $A_z = 0$. Inserting the definition of the canonical momentum we obtain for the final mechanical momentum after the laser pulse has passed:

$$\mathbf{p}_\mathrm{f} = \mathbf{p}_0 + e\mathbf{A}(\eta_0) + \frac{e}{mc}\,\mathbf{p}_0 \cdot \mathbf{A}(\eta_0)\,\hat{z} + \frac{e^2}{2mc}\,\left(\mathbf{A}(\eta_0)\right)^2\,\hat{z}\,.$$

Specifically for a photoelectron with charge -1 and mass 1 in atomic units, one obtains for the momentum gain in z direction due to the propagation in the electromagnetic laser field:

$$p_{\mathrm{f},z} = p_{0,z} + \frac{1}{2c}\,\mathbf{A}(\eta_0) \cdot \left(\mathbf{A}(\eta_0) - 2\mathbf{p}_0\right)\,. \qquad (2)$$

4 Comparison to free electron model within the electric dipole approximation

With the assumption of zero initial momentum p_0 the final momentum in beam propagation direction z becomes proportional to $|\mathbf{A}(\eta_0)|^2$ according to supplementary equation (2). More specifically, for an elliptically polarized laser field with vector potential $\mathbf{A}(t) = A_0 \cdot (\sin(\omega t), -\epsilon\cos(\omega t), 0)$ this leads to a final momentum in beam propagation direction of

$$p_{f,z}(\eta_0) \propto A_0^2 \cdot \left[\sin^2(\eta_0) + \epsilon^2\cos^2(\eta_0)\right]\,. \qquad (3)$$

This is different from the prediction within the electric dipole approximation, where the vector potential $\mathbf{A}$ of the laser field is set spatially homogeneous. Within the electric dipole approximation there is no momentum transfer from the field onto the photoelectron in beam propagation direction (compare Fig. 5).

5 Perturbative treatment of the electron-ion Coulomb interaction

In this section we extend the simple analytic model of the free electron to include the Coulomb interaction between the photoelectron and the residual ion and an initial momentum of the electron in laser beam direction when it appears in the continuum. We study the effect of these two extensions of our model on the ionization phase η_0 for the electron with minimal p_z-shift.

According to [12] the final momentum of the photoelectron can be decomposed into the momentum $\mathbf{p}_\mathrm{L}$ acquired in the laser field and the momentum contribution $\mathbf{p}_\mathrm{C}$ induced by the Coulomb potential of the residual ion

$$\mathbf{p}_\mathrm{final} = \mathbf{p}_\mathrm{L} + \mathbf{p}_\mathrm{C}$$

with $\mathbf{p}_\mathrm{C} \approx \frac{\pi}{4}\frac{t_{1/2}}{r_0^2} \cdot \mathbf{n}_{\mathbf{E}_0}$ and $t_{1/2} = \sqrt{2r_0/|E_0|}$, the time it takes the electron to propagate from the point in space where it enters the continuum to the point in space where the distance to the core has doubled and the influence of the Coulomb-force is reduced to one quarter compared to the tunnel exit. The estimate for $\mathbf{p}_\mathrm{C}$ is obtained by integration of the Coulomb force onto the electron from the residual ion along a straight trajectory that is driven by the constant external electric field E_0 – here approximated as quasi-static – at the moment of ionization:

$$|\mathbf{p}_C| = \int_{t_0}^{\infty} \left.\frac{\partial V(r)}{\partial r}\right|_{r(t)} dt \; , \tag{4}$$

with $V(r) \approx -1/r$ the Coulomb potential of the residual ion. The trajectory described by the photoelectron under the influence of the laser electric field only is given by: $r(t) = \frac{E_0}{2}(t-t_0)^2 + r_0$, assuming that the longitudinal momentum component of the photoelectron vanishes at the tunnel exit $r_0 = r(t_0)$. The integral can be solved analytically and yields:

$$|\mathbf{p}_C| \approx \int_{t_0}^{\infty} \frac{1}{r(t)^2}\, dt = \int_{0}^{\infty} \frac{1}{(r_0 + \frac{E_0}{2}t^2)^2}\, dt = \frac{\pi}{4}\frac{\sqrt{2r_0/E_0}}{r_0^2} = \frac{\pi}{4}\frac{t_{1/2}}{r_0^2} \, . \tag{5}$$

Assuming electric field strengths of $|\mathbf{E}| \ll 1\,\mathrm{a.u.}$, the position for the tunnel exit where the electron appears in the continuum can be approximated as $r_0 \approx \frac{I_\mathrm{P}}{|E_0|}$. $I_\mathrm{p} > 0$ denotes the ionization potential of the target. Accordingly, the Coulomb induced momentum transfer during the ionization process can be rewritten as

$$\mathbf{p}_\mathrm{C} = -\frac{\pi}{2\sqrt{2} I_\mathrm{P}^{3/2}} \left.\frac{\partial \mathbf{A}}{\partial t}\right|_{t=t_0} \, .$$

Since the Coulomb potential induced momentum transfer mainly takes place within a fraction of the first cycle once the electron is in the continuum, we can use $\mathbf{p}_C$ as a perturbative addition to the initial momentum of the electron [13] (see eqs. (1) and (2)) yielding for the final momentum:

$$p_z \approx \frac{1}{2c}\mathbf{A} \cdot [\mathbf{A} - 2\mathbf{p}_0] = \frac{1}{2c}\mathbf{A}(\omega t_0) \cdot \left[\mathbf{A}(\omega t_0) - \kappa \left.\frac{\partial \mathbf{A}}{\partial t}\right|_{t=t_0} \right]$$

with the constant $\kappa = \frac{\pi}{\sqrt{2}} I_{\mathrm{P}}^{-3/2}$. For the second equality in this equation for the final linear momentum p_z we assume that the momentum at the tunnel exit when the electron is born into the continuum is identical to zero. The rotating elliptically polarized laser field is given by $\mathbf{A}(t) = A_0 \cdot (\sin(\omega t), -\epsilon \cos(\omega t), 0)$. We neglect the envelope of the pulse since we are only interested in the dynamics of the electron within a laser optical cycle.

Figure 6 clearly shows the shift of the minimum of the final p_z momentum as a function of the ionization phase towards slightly negative values caused by the Coulomb interaction. Instead of sampling the complete initial momentum space for the electron trajectories, here we only calculated the p_z value for the most probable electron trajectory for each ionization phase, i.e. the electron released with zero transversal momentum at the tunnel exit with respect to the direction of the laser electric field.

Based on this perturbative description of the final photoelectron momentum after the strong-field ionization process we can derive the ionization phase η_0 that leads to the minimum in final p_z momentum analytically:

$$\frac{dp_z}{dt} = 0 \quad \Rightarrow \quad \eta_0 = \omega t_0 = -0.5 \arctan(\omega\kappa) = -0.5 \arctan\left(\frac{\pi}{\sqrt{2}}\, \omega\, I_{\mathrm{P}}^{-3/2}\right) \tag{6}$$

Note that the ionization phase for the electron trajectory with minimal p_z is within this approximation independent of the peak electric field $|E_0|$ and the ellipticity ϵ of the laser pulse.

Furthermore, one can show that the final momentum of the trajectory corresponding to the ionization phase $\eta_0 = \omega t_0$ with minimal p_z points in the direction of the minor axis of the ellipse in the polarization plane, i.e. $p_{x,\mathrm{final}} = 0$.

We can conclude that the angle difference between the streaking angle for the PMD maximum and the streaking angle for the trajectory with the smallest p_z momentum is independent of the laser electric field and by that within this approximation independent of the ellipticity of the driving laser pulse.

This simple model can describe the existence of a minimum in the p_z momentum transfer close to zero ionization phase and predicts the right sign and magnitude for the streaking angle difference between the most probable electron ionized right at the peak of the laser electric field and the electron with minimal linear momentum transfer. However quantitative deviations from the exper-

imental data are to be expected: The model treats the Coulomb induced modification of the final momentum only perturbatively to first order and does not cover any dynamics related to the first step of the two step model of strong-field ionization where the electron is lifted into the continuum.

Supplementary Discussions

Influence of different targets. To study the influence of different targets on the photon linear momentum transfer we have to distinguish between two effects: the modification of the photoelectron dynamics in the polarization plane and the modifications of the photoelectron dynamics in laser beam propagation direction. Whereas the first is significantly influenced by the parent ion interaction, the second is largely independent of the specifics of the ion's potential at long wavelength, i.e. at the mid-IR where our experiments have been performed.

Both can be understood from the semiclassical description of the strong-field ionization process introduced in the previous section. Here the Coulomb (parent ion) interaction is incorporated in the calculations as a modification of the initial momentum $p_0 \approx p_{\mathrm{C}}$ in the polarization plane of the photoelectron at the tunnel exit.

The observed streaking angle offset $\Delta\alpha$ for the minimal photon linear momentum transfer and the most likely electron are directly proportional to the ionization phase offset η_0 and as such in first order to $I_{\mathrm{P}}^{-3/2}$ (compare supplementary equation (6)). The influence of the polarizability of the target has been studied in detail in reference [14] and enters the observation for the streaking angle offset $\Delta\alpha$ similarly as the change of the ionization potential and the related change of the exit radius.

In the equation for the final p_z momentum of the photoelectron the Coulomb interaction enters via the $\frac{1}{c}\mathbf{p}_0 \cdot \mathbf{A}(\eta_0)$ term (supplementary equation (2)). To first order the Coulomb interaction is proportional to the electric field at the moment of ionization, i.e. proportional to the time derivative of the vector potential $\mathbf{A}$, which is by a factor $\omega \approx 0.014\,\mathrm{a.u.}$ (for a central wavelength of $3.4\,\mu\mathrm{m}$) smaller than the magnitude of the vector potential itself. Therefore, the $\mathbf{p} \cdot \mathbf{A}$ is much smaller than the $\mathbf{A}^2$-term in the expression for the final momentum in beam direction and the magnitude of the p_z-shift becomes mostly independent of the specific target.

Even for a reduction of the exit radius, i.e. the distance from the core where the electron appears in the continuum, by a factor of two and a corresponding increase of the p_C term by a factor $\sqrt{0.5}/0.5^2 \approx 2.8$ (compare supplementary equation (5)) the change of the final p_z momentum is on the percent level, well below the resolution of our measurements. Such rather large change of the exit radius corresponds to targets with strongly different ionization potentials probed at the same laser intensity, i.e. xenon ($I_\mathrm{P} = 12.13\,\mathrm{eV}$) and helium ($I_P = 24.587\,\mathrm{eV}$).

We also analyzed the influence of the empirical single active electron potential [15] compared to the bare $-1/r$ Coulomb potential. The relative deviations of the potential are below one percent for the range of interest (radius r larger than the exit radius) for different noble gas targets. This leads to a negligible relative change of the p_C estimate (supplementary equation (5)). The strongest effect is found for the target xenon with two percent relative change of $|\mathbf{p}_\mathrm{C}|$ and therefore again well below the resolution of our measurements.

Initial linear forward momentum. One contribution to the final momentum of the electron not covered by the previous description is the linear momentum transferred to the electron during the tunnel ionization step. Since the electron has to be lifted into the continuum in the presence of the instantaneous laser field additional energy exceeding I_P has to be provided by the absorbed photons. In a cycle-averaged view, this would correspond to the addition of the ponderomotive potential U_p to the effective ionization potential of the target. U_p can be expressed via the vector potential as $\mathbf{A}^2/2$. As the subcycle-resolved version of the ponderomotive potential would be $|\mathbf{A}(\eta)|^2/2$, we suggest a modulation of the initial momentum of the photoelectron momentum in p_z-direction proportional to $\frac{1}{2c}|\mathbf{A}(\eta)|^2$. This corresponds to a momentum transfer related to the energy gain in the laser field. Our measurements suggest a ϵ-dependence of this initial forward momentum in addition to the intrinsic ϵ-dependence from the vector potential. This can be incorporated with the additional prefactor $f(\epsilon)$ – implicitly dependent on ϵ – to the momentum transfer, i.e. $\frac{1}{2c}f(\epsilon)|\mathbf{A}(\eta)|^2$. Without the prefector $f(\epsilon)$ for the initial momentum the minimal momentum transfer – or p_z offset – would become overestimated by up to a factor of two dependent on the ellipticity. For the initial momentum $\mathbf{p}_0 = \mathbf{p}_\mathrm{C} + \frac{1}{2c}f(\epsilon)|\mathbf{A}(\eta)|^2\hat{k}$ of the electron at the tunnel exit we obtain for the ionization phase corresponding to minimal p_z:

$$\eta_0 = -0.5\arctan\left(\frac{\pi}{\sqrt{2}(1+f(\epsilon))}\omega I_\mathrm{P}^{-3/2}\right) .$$

Based on the additional initial linear forward momentum the ionization phase η_0 for photoelectrons with minimal linear momentum transfer p_z becomes dependent on the ellipticity ϵ (compare Fig. 4(b), main text, Model ϵ and Model ϵ^2).

Ellipticity dependence of linear momentum transfer. The analytic model for the final momentum of a free electron born during the laser pulse predicts a clear dependence between the photoelectron momentum in the polarization plane and the longitudinal momentum transfer in laser beam propagation direction (compare supplementary equation (2)). The shift in beam propagation direction is given by $p_z = \frac{1}{2c}\left|\mathbf{A}(\eta)\right|^2$, assuming initial zero momentum. For the momentum in the polarization plane the following holds in first approximation (neglecting the interaction of the photoelectron with the residual ion): $\mathbf{p}_\perp = -\mathbf{A}(\eta)$ where η is the emission phase of the photoelectron. Specifically for the momentum p_{short} along the short axis of the PMD ellipse the following holds: $|\mathbf{p}_{\text{short}}| = |\mathbf{A}(\eta_0)|$. Thus, the expected momentum transfer along the laser beam propagation direction can be expressed as

$$p_z^{\text{expected}} = \frac{1}{2c}\left|\mathbf{p}_{\text{short}}\right|^2 . \tag{7}$$

The comparison in Fig. 3 shows a clearly ellipticity dependent deviation between the measured p_z value and the one calculated following supplementary equation (7). This motivates us together with Fig. 3 from the main text to suggest the additional ϵ-dependent initial photoelectron momentum in laser beam direction at the tunnel exit.

Supplementary Methods

Attoclock technique in non-dipole configuration. The attoclock technique uses the rotating electric field vector of an elliptical or circular polarized laser field as timing reference. This angular streaking technique has so far been successfully used to gain insight into the timing of single and double strong-field ionization processes [14,16–18].

Typically, in the strong-field approximation the parent-ion interaction is neglected and the propagation of the photoelectron in the continuum yields a final momentum of the photoelectron equal to the negative vector potential $-\mathbf{A}$ of the light field at the moment of ionization. This property

connects the timing of an ionization process with the final momentum of the photoelectrons, where the electric field vector serves as timing reference. Strong-field ionization dominantly takes place around the maximum of the electric field. And thus for elliptical polarization one observes maxima of the electron signal mainly along the short axis of an ellipse-shaped electron momentum distribution. The parent-ion interaction and possible ionization delay times lead to an angle offset of the position of the maximum of the photoelectron momentum distribution in the polarization plane. The electron dynamics due to the laser electric field is mainly taking place in the polarization plane (p_x-p_y-plane). Within the electric dipole approximation the 3D PMD from strong-field ionization is symmetric with respect to the polarization plane ($p_z = 0$).

A long wavelength driving laser field can accelerate the photoelectrons to velocities where magnetic field effects become significant already for relatively low field strength due to the $\mathbf{v} \times \mathbf{B}$ term of the Lorentz force. This leads to a modulation in p_z-direction, i.e. along the laser propagation direction. However, the timing reference is still provided by the electric field linked to the electron momentum distribution in the polarization plane.

Here, we use the angular information in the polarization plane to study the momentum shift in positive p_z direction as a function of the ionization phase within the laser optical cycle.

We use elliptical coordinates in the polarization plane (instead of typically used polar coordinates) to ensure a linear mapping between the streaking angle and ionization time not only for circular polarization but also for small ellipticities.

In our experiment, we have contributions not only from the dominant central cycle, but also from neighbouring cycles (compare Fig. 1, main text). This results in an effective intensity averaging as the peak electric fields of the neighbouring cycles are lower.

In the final PMD we cannot distinguish the contributions from the various cycles with a similar peak electric field since the electrons emitted at a certain phase end up at the same angle. Thus, the only influence of the contributions from several optical cycles is an effective peak intensity averaging.

Polarimetry. We use optical polarimetry measurements to determine the polarization state of the laser pulse [19]. The laser beam passes through a custom made MgF_2 quarter- and half-wave plate (B.Halle) for the polarization control and subsequently a wire grid polarizer (thorlabs WP25M-UB) with a high extinction ratio in the mid-IR spectral region ($> 10000 : 1$). The transmitted laser intensity after the polarizer is measured with a lock-in amplifier as a function of the half-wave plate orientation α (angular precision: $< 1\,\mathrm{mrad}$). It follows the analytic expression

$$\begin{aligned} I^{\mathrm{pol}}(\alpha) &= I_0^{\mathrm{pol}}(\epsilon) \cdot [\cos^2(2\alpha - \alpha_0) + \epsilon^2 \cdot \sin^2(2\alpha - \alpha_0)] \\ &= I_0^{\mathrm{pol}}(\epsilon) \cdot [1 + \left(\epsilon^2 - 1\right) \cdot \sin^2(2\alpha - \alpha_0)] \end{aligned}$$

with $I_0^{\mathrm{pol}}(\epsilon)$ being the ellipticity dependent maximal intensity measured behind the polarizer. From the fit we extract the ellipticity ϵ and the orientation α_0 of the major axis of the polarization ellipse with respect to the orientation of the polarizer.

The exact orientation of the polarization ellipse in the VMI spectrometer is then found from a reference measurement at linear polarization.

Definition of the elliptical coordinate system. PMDs from strong-field ionization recorded with elliptically polarized laser pulses are typically ellipse-shaped due to the close relation between the final momentum of the photoelectron $\mathbf{p}_f$ and the vector potential $\mathbf{A}(t_0)$ at the time of ionization t_0, $\mathbf{p}_\mathrm{f} \approx -\mathbf{A}(t_0)$. This connection suggests elliptical coordinates as a suitable choice for our analysis. We define the elliptical coordinates similar to [20] but ensure a mathematically self consistent mapping also for small ellipticities using curved isolines for the angle coordinate. The mapping between the momentum in Cartesian coordinates $\mathbf{p} = (p_x, p_y, p_z)$ and the momentum in elliptical coordinates in the polarization plane $\mathbf{p} = (p_\alpha, p_\rho, p_z)$ is given by

$$\begin{aligned} p_\alpha &= \mathrm{Im}\left\{\cosh^{-1}\left(\tfrac{1}{f}(p_x - ip_y)\right)\right\} \\ p_\rho &= \mathrm{Re}\left\{\cosh^{-1}\left(\tfrac{1}{f}(p_x - ip_y)\right)\right\} \\ p_z &= p_z \end{aligned}$$

The parameter f giving the distance of the focal point of the ellipse to the origin is linked to

the ellipticity $\epsilon = \frac{a}{b}$ by $f = \sqrt{b^2 - a^2}$, while a and b are the short and long axis of the ellipse, respectively. The domains of the elliptical coordinates are $p_\alpha \in (-\pi, \pi]$ and $p_\rho \in [0, \infty)$.

The inverse mapping is as follows:

$$\begin{aligned} p_x &= f \cosh p_\rho \cos p_\alpha \\ p_y &= -f \sinh p_\rho \sin p_\alpha \\ p_z &= p_z \end{aligned}$$

In the limit of $\epsilon \to 1$ the elliptical coordinates transform into polar coordinates with p_α corresponding to the angle coordinate and p_ρ to the radial coordinate. The elliptical coordinate system is illustrated in supplementary figure 1.

The mapping between the elliptical angle coordinate α and the time t is directly linear. To show this we compare the two parametrizations for the electric field in the polarization plane neglecting the pulse envelope. Parameterized by time t the electric field reads:

$$\begin{aligned} E_x &= \frac{E_0}{\sqrt{1+\epsilon^2}} \cos(\omega t + \phi_0) \\ E_y &= \epsilon \frac{E_0}{\sqrt{1+\epsilon^2}} \sin(\omega t + \phi_0) \,. \end{aligned}$$

The phase offset ϕ_0 in the trigonometric functions can be set to zero without loss of generality. Parameterized in terms of the elliptical coordinates angle α the electric field is given by:

$$\begin{aligned} E_x &= a \cosh E_\rho \cos \alpha \\ E_y &= a \sinh E_\rho \sin \alpha \end{aligned}$$

Matching both parametrizations for the electric field yields:

$$\begin{aligned} \frac{E_0}{\sqrt{1+\epsilon^2}} \cos(\omega t) &= a \cosh E_\rho \cos \alpha \\ \epsilon \frac{E_0}{\sqrt{1+\epsilon^2}} \sin(\omega t) &= a \sinh E_\rho \sin \alpha \end{aligned}$$

We divide the lower equation by the upper one and end up with:

$$\epsilon \tan(\omega t) = \tanh(E_\rho) \tan \alpha$$

With the identification $\epsilon = \tanh(E_\rho)$ this shows the linear mapping between time t and angle α.

Extraction of the p_z-offset and the PMD signal as function of the streaking angle α. To extract the linear momentum transfer from the laser field onto the photoelectron during the ionization process as a function of the streaking angle α we start from the reconstructed 3D PMD in Cartesian coordinates. We map this 3D PMD onto the elliptical coordinates. To determine the correct focal point parameter f for the mapping at a given set of laser parameters we use the ellipticity extracted from optical polarimetry (see section polarimetry) and the short axis of the polarisation ellipse. The latter we extract from the 3D PMD with a double Gaussian fit to the PMD slice $|p_x| \leq 0.1\,\mathrm{a.u.}$ projected onto the p_y-axis.

To suppress possible background in the signal free region of the PMD we restrict p_ρ in the polarization plane to $p_\rho \in [p_{\rho,min}, p_{\rho,max}]$ with

$$\begin{aligned} p_{\rho,min} &= \mathrm{Re}\left\{\cosh^{-1}\left(-\tfrac{i}{f}(a - 2.2\,\sigma_a)\right)\right\} \\ p_{\rho,max} &= \mathrm{Re}\left\{\cosh^{-1}\left(-\tfrac{i}{f}(a + 2.2\,\sigma_a)\right)\right\}. \end{aligned}$$

σ_a is the radial width of the PMD, i.e. the standard deviation of the double Gaussian fit used to extract the short axis a of the polarization ellipse.

The PMD mapped to elliptical coordinates is integrated along the p_ρ direction and binned in p_α between $-\pi$ and π into bins of width $\pi/180$. This yields the PMD as a function of the angle coordinate p_α and the momentum in beam propagation direction p_z.

We extract the shift of the PMD in p_z direction as a function of the streaking angle with a Gaussian fit to each slice of the 2D PMD (p_α, p_z) at a fixed p_α (Fig. 2). We choose a Gaussian fit function to match the typical description of the momentum distribution of the photoelectron at the tunnel exit perpendicular to the electric field direction at the moment of ionization.

In Fig. 3 of the main text we show the points for angles with sufficiently high statistics for the Gaussian fit (coefficient of determination $R^2 \geq 0.95$). The exact angular position α_M of the minimum of the p_z-offset is determined from the fit with the analytic expression

$$\begin{aligned} p_z(\alpha) &= c_1 \cdot \left[\epsilon^2 \cos^2(\alpha - \alpha_\mathrm{M}) + \sin^2(\alpha - \alpha_\mathrm{M})\right] + c_2 \\ &= c_1 \cdot (1 - \epsilon^2) \cdot \sin^2(\alpha - \alpha_\mathrm{M}) + \tilde{c}_2\,, \end{aligned} \tag{8}$$

to the p_z-offset as a function of α considering the errors of the previous Gaussian fits as inverse weighting factors. Here ϵ is the ellipticity obtained from optical polarimetry and c_1, c_2 and $\tilde{c}_2$ are

strictly positive fitting parameters. The choice of this fit function is motivated by the analytic expression for the final momentum in beam propagation direction $p_z \propto |\mathbf{A}(\eta_0)|^2$ of the photoelectron following supplementary equation (2).

The streaking angle α_{I} of maximal PMD signal is extracted from the 2D PMD (p_α, p_z) via projection onto p_z and a subsequent double Gaussian fit with fixed phase difference π:

$$f(p_\alpha) = d_1 \left[\exp\left(-d_2 \left(p_\alpha - \alpha_{\mathrm{I}} - \frac{\pi}{2} \right)^2 \right) + \exp\left(-d_2 \left(p_\alpha - \alpha_{\mathrm{I}} + \frac{\pi}{2} \right)^2 \right) \right] + d_3 \tag{9}$$

The fitting parameters d_1, d_2, d_3 are real and strictly positive.

Experimental uncertainties and error propagation. The recorded 2D projections of the 3D PMD are time-integrated photoelectron ensembles. Although single hit assignment in the measurement is not possible due to the high repetition rate of the laser and the readout time of the CCD camera, the electronic and readout noise of the CCD camera are far below the strength of an actual signal ($< 10^{-3}$), allowing for a nevertheless good signal to noise ratio.

The error bars we show in Fig. 3 of the main text correspond to the 1σ uncertainty from the gaussian fit for $\langle p_z \rangle$ at fixed angle α, i.e. the uncertainty of the estimator $\widehat{\langle p_z \rangle}$ for the peak position or mean value of the Gaussian. The error bars in Fig. 3 do not include any systematic errors for example for the zero momentum position on the detector.

The 1σ error bars $\sigma_{\Delta\alpha}$ shown in Fig. 4(b) of the main text are calculated as following:

$$\sigma_{\Delta\alpha} = \sqrt{\sigma^2_{\alpha_{\mathrm{I}}} + \sigma^2_{\alpha_{\mathrm{M}}}}\,.$$

$\sigma_{\alpha_{\mathrm{I}}}$ is the 1σ uncertainty on the α_{I} value from the fit with supplementary equation (9). $\sigma_{\alpha_{\mathrm{M}}}$ is the 1σ uncertainty on α_{M} extracted from the fit with supplementary equation (8) using the 1σ uncertainties for the $\langle p_z \rangle$ at fixed angle α as inverse weighting factors.

Note that a systematic error for the zero momentum calibration of the detector would not enter the two extracted angles α_{I} and α_{M}. Additional systematic errors, e.g. in the zero calibration of the streaking angle α cancel out in the reported angle differences $\Delta\alpha$.

Supplementary References

1. Smeenk, C. T. L. *et al.* Partitioning of the linear photon momentum in multiphoton ionization. *Phys. Rev. Lett.* **106**, 193002 (2011).

2. Chelkowski, S., Bandrauk, A. D. & Corkum, P. B. Photon momentum sharing between an electron and an ion in photoionization: From one-photon (photoelectric effect) to multiphoton absorption. *Phys. Rev. Lett.* **113**, 263005 (2014).

3. Klaiber, M., Yakaboylu, E., Bauke, H., Hatsagortsyan, K. Z. & Keitel, C. H. Under-the-barrier dynamics in laser-induced relativistic tunneling. *Phys. Rev. Lett.* **110**, 153004 (2013).

4. Van den Heuvell, H. V. L. & Muller, H. Multiphoton processes. edited by S. J. Smith and P. L. Knight. *Cambridge Univ. Press, Cambridge* (1988).

5. Gallagher, T. Above-threshold ionization in low-frequency limit. *Phys. Rev. Lett.* **61**, 2304 (1988).

6. Schafer, K., Yang, B., DiMauro, L. & Kulander, K. Above threshold ionization beyond the high harmonic cutoff. *Phys. Rev. Lett.* **70**, 1599 (1993).

7. Corkum, P. B. Plasma perspective on strong field multiphoton ionization. *Phys. Rev. Lett.* **71**, 1994–1997 (1993).

8. Yudin, G. L. & Ivanov, M. Y. Nonadiabatic tunnel ionization: Looking inside a laser cycle. *Phys. Rev. A* **64**, 013409 (2001).

9. Geng, J.-W. *et al.* Nonadiabatic tunneling ionization of atoms in elliptically polarized laser fields. *J. Phys. B: Atomic, Molecular and Optical Physics* **47**, 204027 (2014).

10. Lai, Y. H. *et al.* Experimental investigation of strong-field-ionization theories for laser fields from visible to midinfrared frequencies. *Physical Review A* **96**, 063417 (2017).

11. Mur, V. D., Popruzhenko, S. V. & Popov, V. S. Energy and momentum spectra of photoelectrons under conditions of ionization by strong laser radiation (the case of elliptic polarization). *JETP* **92**, 777–788 (2001).

12. Goreslavski, S. P., Paulus, G. G., Popruzhenko, S. V. & Shvetsov-Shilovski, N. I. Coulomb asymmetry in above-threshold ionization. *Phys. Rev. Lett.* **93**, 233002 (2004).

13. Bardsley, J. N., Penetrante, B. M. & Mittleman, M. H. Relativistic dynamics of electrons in intense laser fields. *Phys. Rev. A* **40**, 3823–3835 (1989).

14. Pfeiffer, A. N. *et al.* Attoclock reveals natural coordinates of the laser-induced tunnelling current flow in atoms. *Nat. Phys.* **8**, 76–80 (2012).

15. Milošević, D. B. *et al.* Strong-field electron spectra of rare-gas atoms in the rescattering regime: enhanced spectral regions and a simulation of the experiment. *J. Phys. B* **43**, 015401 (2009).

16. Eckle, P. *et al.* Attosecond ionization and tunneling delay time measurements in helium. *Science* **322**, 1525–1529 (2008).

17. Landsman, A. *et al.* Ultrafast resolution of tunneling delay time. *Optica* **1**, 343–349 (2014).

18. Hofmann, C., Landsman, A. S. & Keller, U. Attoclock revisited on electron tunnelling time. *J. Mod. Opt.* **66**, 1052–1070 (2019).

19. Weger, M., Maurer, J., Ludwig, A., Gallmann, L. & Keller, U. Transferring the attoclock technique to velocity map imaging. *Opt. Expr.* **21**, 21981–21990 (2013).

20. Hofmann, C., Landsman, A. S., Cirelli, C., Pfeiffer, A. N. & Keller, U. Comparison of different approaches to the longitudinal momentum spread after tunnel ionization. *J. Phys. B* **46**, 125601 (2013).
